# An Efficient Data Acquisition and Processing Scheme for Wireless Multimedia Sensor Networks

**DOI:** 10.1155/2022/6394029

**Published:** 2022-07-13

**Authors:** Kun Gao, Hao Wang, Joanicjusz Nazarko

**Affiliations:** ^1^Zhejiang Business Technology Institute, Ningbo 315012, China; ^2^Ningbo Tech University, Ningbo 315012, China; ^3^Bialystok University of Technology, 15-351 Bialystok, Poland

## Abstract

This study studies the problem of efficient multimedia data acquisition and decreasing whole energy expenditure of wireless multimedia sensor networks and proposes a three-step multimedia data acquisition and wireless energy supplement strategy. Firstly, for network partition, this study proposes a network partition scheme based on vicinity likeness and distance of sensor nodes (VLD), which divides the whole sensor network into multiple regions. The physical links inside the region are dense and concentrated, while the link connections between regions are sparse. Disconnecting the connections between regions hardly affects the data transmission of sensor nodes. Secondly, this study proposes an efficient data acquisition and processing scheme for wireless multimedia sensor network ASS. Compared with other anchor selection schemes, this scheme has obvious performance advantages. Then, the problem of minimizing network energy expenditure is defined, and the optimal sensor node data perception rate and network link transmission rate of the optimization function are obtained by dual decomposition and sub-gradient method. Finally, in the case of a given network energy threshold, the performance of the overall strategy in this study is verified by comparing the amount of data collected by the base station.

## 1. Introduction

Wireless multimedia sensor networks (WMSNs) [1] are usually multimedia sensor nodes with computing, storage, and communication capabilities. They are self-organized distributed perception networks capable of sensing, collecting, processing, and transmitting multimedia information such as audio, video, static images, and numerical data within the coverage area of the network. Data acquisition has always been one of the key research issues in WMSNs. The energy used by sensor nodes to sense, receive, and transmit states comes from energy acquisition module and energy storage module. The data transmitted by nodes are closely related to battery energy. To prolong the life of rechargeable nodes as much as possible and overcome the problems of unstable energy source and insufficient density obtained by sensor nodes from the surrounding environment using energy collection technology, wireless power transmission (WPT) technology provides a new method to solve the problem of battery energy limitation. Its energy is more stable and controllable, and the charging time for rechargeable sensor nodes is shorter. In the process of energy transmission, because the energy density decreases with the increase in distance, to improve the energy efficiency and node survival rate, the use of a mobile car equipped with energy transmission equipment, namely wireless energy supplement machine (WESM), can effectively reduce the charging waiting time of sensor nodes, improve the energy receiving density, and prolong the service life of rechargeable sensor networks [2–5]. Compared with deploying static charger, WESM can better adapt to the changes in network topology and better manage the sensor anchor.

For the problem of data transmission in WMSNs, most research work generally uses multi-hop form [6–9] or data acquisition machine (DAM) to transmit the data sensed by sensor nodes to the base station. In the process of transmitting data in the form of multi-hop, the sensor node not only needs to undertake the task of its own data sensing and transmission but also needs to receive and forwards the data packets of other neighbor nodes, so that the energy expenditure rate of the sensor node near the base station or anchor point is higher than that of other nodes, which quickly reduces its service life, which is easy to lead to service interruption and energy hotspot problems. To alleviate the problems of energy expenditure and data acquisition delay of sensor nodes, this study proposes to use the combination of multi-hop transmission and DAM to solve the data acquisition problem of rechargeable network [10–12].

The combination of WESM and DAM can not only supplement energy for rechargeable sensor nodes and prolong the network life but also effectively balance the node communication energy expenditure and data acquisition delay. To avoid the one-way decline of the battery sensor anchor, this study uses WESM to supplement energy for short distance, so as to achieve the purpose of long-term use of nodes and unlimited extension of network life. DAM and WESM move in the sensing area, which are, respectively, responsible for collecting data at the anchor point and supplementing energy for the nodes to be charged, to prolong the service life of the network as much as possible. The aim of this study was to design an efficient and low-energy expenditure data acquisition and wireless energy supplement strategy, so that the network can efficiently obtain sensor node data and reduce network energy expenditure.

This study presents a three-step method for data acquisition and wireless energy supplement design. When the range of sensor network is large, to reduce the data acquisition delay of sensor nodes, firstly, the network is divided into multiple subareas, and the responsibility range of each DAM and WESM is determined; that is, the network area is divided; the second step is to determine the moving path of DAM in each subarea, that is, the selection of anchor points; finally, we study how to achieve the optimal network performance when DAM cruises at each anchor point. In this study, the optimal network performance is defined as the problem of minimizing network energy expenditure. We can achieve the purpose of minimizing network energy expenditure by the optimal node perception rate and physical link transmission rate.

The organizational structure of this study is as follows: Section 1 of this study describes the relevant research.  Section 2 introduces the network model and network partition scheme VLD.  Section 3 designs the anchor point selection scheme ASS based on node sociality and energy. Next, in Section 4, the network energy expenditure function is solved by duality theory to obtain the optimal node perception rate and physical link transmission rate, so as to minimize the network energy expenditure.  Section 5 verifies the performance of the proposed scheme VLD, ASS, and the overall strategy of this study through experimental analysis. Finally, Section 6 summarizes this study.

## 2. Relevant Research

This section classifies and summarizes the existing work according to the three steps of designing multimedia data acquisition and wireless energy supplement strategy in wireless multimedia sensor networks, that is, network zoning scheme, adaptive anchor selection scheme, and network optimization goal.

### 2.1. Network Partition Scheme

To achieve a high amount of DAM data acquisition and WESM charging efficiency, the sensor network is usually divided into multiple subareas according to the distance between sensor nodes, physical link connection, and the deployment direction of base stations.

Reference [13] uses multiple WESMs to supplement energy for sensor nodes, proposes to divide the sensor network into multiple subregions, and deploys a WESM in each subregion to supplement energy for nodes in the region. This study proposes an adaptive zoning scheme. Firstly, the sensor node with the least energy is selected as the central node of each subregion after zoning, and all nodes are assigned to the nearest central node. The coordinates of each sub-node to the center of all sub-nodes are reassigned according to the Cartesian distance of each node. This process is repeated until all central nodes in the network are no longer changed. Reference [14] proposed a division algorithm. In this method, the sensor network is divided into internal and external parts: the sensor nodes centered on the base station and within *k*-hops from the base station are used as inner nodes; the remaining nodes act as external nodes and use DAM to obtain node data. Considering the zoning method of complex social networks and adopting the characteristics that complex social networks obey normal distribution, [15] proposed the minimum cut set algorithm and subnet generation algorithm based on normal distribution, so that the subnet has similar node degree distribution with the original network, which greatly reduces the computational complexity of dynamic online social networks. Reference [16] designed a scheme of multimedia data acquisition, which is responsible for the data acquisition and energy supplement process of sensor nodes. According to the number of MC, this study proposes a two partition algorithm based on center points (TP-CP), which divides the sensor network into multiple regions; that is, firstly, the network is initially divided and the central node of the subregion is selected; then, through the distance from all other sensor nodes to the central node and the static routing length, the node is divided into one of the central nodes until all nodes have their own partitions.

### 2.2. Adaptive Anchor Point Selection Scheme

To reduce the communication energy expenditure and data acquisition delay of sensor nodes, it is usually necessary to consider how to select the data acquisition anchor point in the network and build the shortest mobile path of DAM.

Reference [13] proposes to cover the sensing area with a series of circles. The center length of any two adjacent circles is (3)^1/2^*kd*_*r*_, where *k* represents the coverage of the anchor point, and *k*-hop and *d*_*r*_ represent the sensing range of the sensor node. The center of each circle is the data acquisition anchor point in the network. Then, DAM constructs the shortest traveling salesman problem (TSP) path of all anchor points to obtain data at the anchor points. Reference [16] proposes an anchor selection algorithm based on neighbor amount and residual sensor anchor (AS-NAE). The reference selects the anchor based on the k-hop and the minimum electric quantity of nodes in the region and then constructs the TSP path of the anchor in the region. DAM patrols the path and collects node data. Reference [17] arranges the minimum neighbor node power of all nodes in ascending order and selects the node with the maximum power as the anchor. Reference [18] constructs the weight of each node by calculating the number and power of *k*-hop neighbor nodes of sensor nodes and successively selects the node with the largest weight as the data acquisition anchor.

### 2.3. Network Optimization Objectives

At present, network lifetime, system energy expenditure, data acquisition volume, and data acquisition delay are usually taken as the system optimization objectives, and the optimization function is constructed according to these indicators to obtain the optimal performance of the system.

Aiming at the problem of network lifetime, [19] analyzes the energy expenditure of wireless multimedia sensor networks. The wireless sensors in the network have adjustable sensing range. This study also proposes a node deployment strategy to meet the minimum network lifetime *T*, which greatly improves the node survival rate. Reference [20] designed a deployment strategy of directed charger to improve the charging efficiency and network life of sensor networks. Reference [21] proposed an analytical model to judge the area where hot issues occur and predict the remaining time of the network. On this basis, they apply the temporal and spatial changes in energy hotspots to network routing, which greatly balances the energy expenditure of sensor nodes and improves the service time of the network. Reference [22] proposed a dynamic routing scheme based on ring. Aggregating data in non-hotspot areas improves the network life cycle.

Aiming at the problem of system energy expenditure, [11] designs the acquisition path by calculating the heuristic algorithm to balance the transmission load in multi-hop transmission, so as to reduce the overall energy expenditure of the system. Reference [23] uses the mobile base station to obtain data of each anchor point and designs the optimal mobile path of the mobile base station, which effectively reduces the mobile energy expenditure of data acquisition by DAM. Starting from the system energy expenditure, [24] analyzes the energy expenditure model and the data acquisition model of DAM and constructs the energy expenditure optimization functions of nodes and DAM, respectively, to obtain the optimal data transmission volume of nodes and the residence time of DAM at each anchor point.

To solve the problem of data acquisition volume, [25] proposed a two-tier architecture model under the condition of limited resources such as link bandwidth and node energy, to maximize the data transmission rate from all nodes to the collector, so as to improve the data acquisition volume in the network. Reference [14] uses a DAM to complete the data acquisition process of the whole network. The node data packets are finally transmitted to the base station in two ways: multi-hop transmission and mobile element collection. By designing the minimum spanning tree from the node to the anchor point or base station, the communication energy expenditure and trolley mobile energy expenditure are reduced, the network data are collected effectively, and the energy hot spot problem is alleviated. Reference [17] uses a mobile car to obtain sensor node data and wirelessly charge the nodes and designs an optimization function about the amount of data acquisition, which greatly improves the data acquisition performance of the network. However, because the charging time cannot be ignored, the nodes need to wait a long time for the data to be collected, which increases the data collection delay.

To solve the problem of data acquisition delay, [13] constructs the TSP paths of the anchor point and the node to be charged, respectively, uses DAM to obtain the data at the anchor point, and uses WESM to charge the nodes in the network, which will greatly reduce the waiting time for data acquisition. Starting from reducing the network patrol path, [10] designed a heuristic algorithm to gradually reduce the moving path and data acquisition delay of DAM. Reference [26] proposed an optimization problem based on dynamic topology with the goal of maximizing the dwell time ratio of energy supply equipment, that is, minimizing the ratio of mobile time and service time between wireless energy supply and data acquisition equipment. By analyzing the working constraints of nodes and equipment, the original problem was transformed into a multistate linear programming problem, which effectively reduced the data acquisition delay and improved the network operation time. Reference [27] designed a broadcast protocol combining negative answer and acknowledgment frames, which greatly improved the network operation cycle and reduced the data transmission delay.

### 2.4. Analysis and Comparison

The novelty of this study lies in the following:

- Firstly, compared with the existing work, this study proposes a novel sensor network partition scheme, which comprehensively considers the vicinity likeness of sensor nodes and the distance between nodes. In the existing work, the partition scheme is based on multiple WESMs or only considers the distance and routing of nodes and does not consider the impact of multiple DAMs and WESMs and the neighborhood nodes of nodes on the partition network.
- Secondly, because most current anchor selection schemes do not analyze the influence of sensor node sociality and k-hop neighbor energy on anchor selection at the same time, this study considers the influence of both and designs a new adaptive anchor selection scheme.
- Finally, aiming at minimizing the system energy expenditure, this study calculates the optimal data sensing rate and link transmission rate of sensor nodes to achieve the optimal performance of the network.

**Figure 1:**
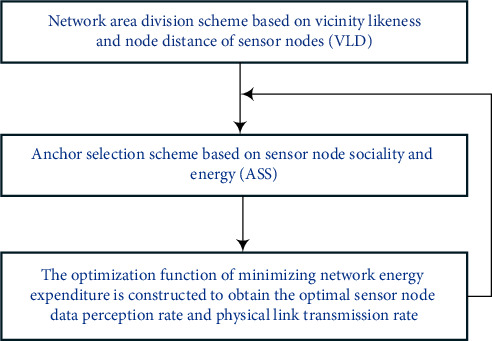
Network frame diagram.

Figure 1 shows the overall network framework of the strategy in this study. According to the coordinates and routing information of sensor nodes, this study designs an VLD scheme, which divides the network into multiple subregions and obtains the connection of nodes in each subregion. Then, when the sensor node connection and battery energy are known, the ASS scheme is used to determine the anchor point and its coverage node in each area, as well as the optimal transmission path from the sensor node data to the anchor point. Finally, the dual theory and gradient method are used to solve the optimization function of network energy expenditure, so as to obtain the optimal node data perception rate and link transmission rate and realize the optimal network performance. When DAM completes a round of data acquisition process, because the data transmitted by the sensor node will dynamically change the node residual energy, to achieve better data acquisition results, this strategy will reselect the anchor point in each subarea according to the node residual battery energy.

## 3. Overview of System Model

### 3.1. Network Model

In the network area where *n* rechargeable sensor nodes are evenly distributed, the radio frequency (RF) energy sent from WESM can be stored in the energy storage device (rechargeable battery) for data sensing and transmission.

If two sensor nodes can transmit data below the maximum transmission power, there is a physical link between the two nodes. The maximum transmission range of a node depends on its maximum transmission power. Only nodes within the transmission range can communicate directly with neighbor nodes [28]. In this study, whether there is a physical link between sensor nodes is determined by its sensing range. Without considering the influence of sensor node deployment terrain, once the Euclidean distance between two sensor nodes is less than the sensor range of the node itself, it is considered that there is a physical link. In this network model, any two nodes can be connected through at least one-hop routing, and there are no sensor nodes that cannot communicate with each other in the network.

**Figure 2:**
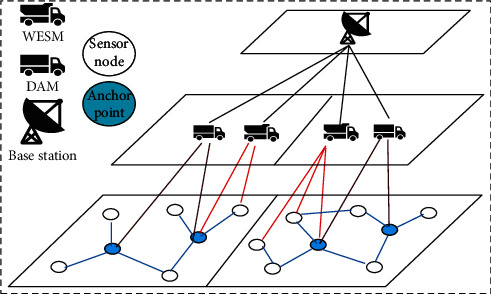
Network scenario.

Considering the WMSNS scenario with multiple DAMs and WESMs, the whole sensor network architecture is divided into three layers, as shown in Figure 2.

Each layer contains different elements in the network.

- The bottom layer is used to deploy all sensor nodes and select some nodes as anchor points. The sensor node transmits the statically sensed data to the anchor point in the form of multi-hop.
- The middle layer is a series of resource-rich mobile cars, DAM and WESM. Through the network partition algorithm, the network is divided into multiple areas, and a WESM and DAM car are deployed in each area. WESM starts from the station, patrols the whole area, and finally returns to the starting point to wirelessly charge the sensor node sending the charging request. In this process, the DAM in the area periodically patrols each anchor point, collects the data at the anchor point, and transmits the collected data to the base station.
- The top layer is used to place the base station, which is located in the center of the whole network area. It is used to process the collected data and charge the mobile car to maintain the continuous operation of the whole network.

### 3.2. Network Partition Scheme

Compared with static charging pile and multi-hop communication of the whole network, the use of mobile charging node can greatly enhance the charging process and controllability, and the use of multimedia data acquisition car can effectively reduce the communication energy expenditure and delay. However, when the network range is large, WESM may not be able to move to the location of the sensor node to be charged in time or complete the charging queue, resulting in the death of the sensor node and the interruption of the network. In addition, multiple DAMs may be concentrated in the same area, which affects the overall network performance. To improve the data acquisition of DAM and the charging efficiency of WESM, this study proposes a network area division scheme based on the vicinity likeness of sensor nodes and the distance between nodes, namely VLD. The scheme divides the network into multiple regions and deploys a DAM and WESM in each region, which are, respectively, responsible for data acquisition and power supplement of sensor nodes in the region.

The vicinity likeness of sensor nodes was first proposed by reference [29], which is used to measure the similarity between any two objects in the topology map. Due to the high time complexity of calculating the similarity of sensor nodes, [30] proposed an efficient SimRank calculation method based on local information redundancy, which reduces the time complexity from *O*(*k*|*e*|^2^) to *O*(*k*|*e*|*v*|), where *k* is the total number of iterations, *e* is the number of edges, and *V* is the number of nodes.

The basic idea of SimRank model is as follows: if two sensor nodes have similar neighborhoods in the graph-based topology, that is, there are more similar neighbor nodes, then the two sensor nodes should also be similar. Therefore, whether two sensor nodes are similar is determined by their neighbor nodes. When the sensing range of sensor nodes is large and the distribution of nodes in the network is dense, it is far from enough to rely on the vicinity likeness between nodes. Therefore, this study also takes the distance between sensor nodes as an important index to measure whether nodes belong to the same partition.

Firstly, the similarity between the sensor node and itself is defined as 1. For sensor nodes *i* and *j* with neighborhood, their similarity is defined as the mean of the pairwise similarity of all their one-hop neighbors and then multiplied by the damping coefficient C. For any two sensor nodes *i* and *j* with neighbor nodes, the similarity between sensor nodes is expressed as follows:

(1) $$\begin{array}{c} r\left(i,j\right)=\left\{\begin{array}{cc} 1, & i=j,\\ \frac{c}{N_{1\_\text{hop}}\left(i\right)N_{1\_\text{hop}}\left(j\right)}\sum_{m,l}r\left(m,l\right), & i\neq j. \end{array}\right. \end{array}$$

Here, sensor node *m* ∈ *N*_1_hop_(*i*), *l* ∈ *N*_1_hop_(*j*).*N*_1_hop_(*i*) represents the set of vicinity nodes of sensor node *i* in the first hop; *r* (*m*, *l*) represents the similarity between node *m* and node *l*; and *c* is a damping coefficient, so that the farther the distance between two sensor nodes, the smaller the influence on *r* (*i*, *j*).

If there are sensor nodes *i* and *j*, and *N*_1_hop_(*i*) = Ø or *N*_1_hop_(*j*) = Ø, then *r* (*i*, *j*) = 0. Using matrix *R* to represent the pairwise similarity matrix of sensor nodes in the network, then matrix *R* is a symmetric matrix with diagonal element of 1.

If the distance between any two sensor nodes is represented by matrix *D*, then matrix *D* is a symmetric matrix with diagonal 0. The lower triangular element values are sorted in matrices *R* and *D* from small to large and number them from 1, and then, the numbers are put into the position in the matrix where the corresponding element values are located. Matrix *B* is defined as the partition index of sensor nodes *i* and *j*, and then, *B* is expressed as follows:

(2) $$\begin{array}{c} B=\left\{\begin{array}{cc} 0, & i⩽j,\\ 0.5R\left(i,j\right)+0.5\;D\left(i,j\right), & i>j. \end{array}\right. \end{array}$$

Then, according to the hierarchical clustering method, the two sensor nodes with the smallest value in the triangle element under matrix B are divided into the same region until the whole network region is divided into the given H part.

**Figure 3:**
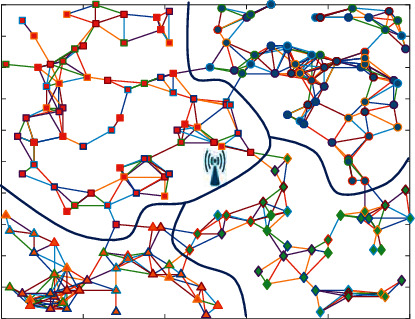
Example diagram of network partition.

200 rechargeable sensor nodes are set evenly distributed within 100 m × 100 m sensing area. The sensing range of each sensor node is *D*_*r*_ = 10 m. The partition example results of network partition scheme VLD are shown in Figure 3. The whole network is divided into four parts. It can be seen from the figure that all nodes in the network can reach, and the physical links in the same partition are dense, and the links between partitions are sparse. Disconnecting the link connection of sensor nodes between partitions will hardly affect the transmission of node packets.

After the network area is divided, a DAM and WESM are placed in each area to ensure data acquisition and node energy supplement. The sensor node with the smallest sum of distances to other sensor nodes and the sum of routing hops in each area as the starting point of the trolley are selected [16], that is, the station.

## 4. Multimedia Data Acquisition

### 4.1. Adaptive Anchor Point Selection Scheme

To make the communication energy transmitted from sensor nodes to base stations run out as little as possible, this study selects some sensor nodes in the region as anchor points to obtain the data generated by nodes in their clusters. Because the anchor point needs to send and receive data from other sensor nodes frequently, it brings a great burden on the battery cost of the node. Therefore, the sensor nodes with large battery energy and as many neighbor nodes as possible should first be selected as the anchor point, that is, the data acquisition point of DAM.

The number of neighbor nodes is closely related to the sociality of sensor nodes. To calculate the sociality of each sensor node in the partition, the number of neighbor nodes in the *k*-hop static route of the sensor node in the region is indicated by defining the connectivity matrix *X*. If the sensor node *i* and node *j* are reachable within *k*-hops, *X*_*ij*_ = 1; otherwise, *X*_*ij*_ = 0. In addition, the diagonal element of matrix *X* is set to 0, which represents that there is no self-circulation in the network. *N*^*h*^ represents the number of sensor nodes in area *h*, and the density in this area *ρ*_*i*_ is as follows:

(3) $$\begin{array}{c} \rho_{i}=\frac{\left|\cup \sum_{s=1}^{k}N_{s\_\text{hop}}\left(i\right)\right|}{N^{h}}, \end{array}$$

where *N*_*s*_hop_(*i*) represents the set of neighbor nodes of sensor node *i* in *s*-hop. Then, the minimum battery energy batt (*i*) of the neighbor node of the sensor node *i* within *k*-hops is calculated, and its value is given as follows:

(4) $$\begin{array}{c} \text{batt}\left(i\right)=\left\{\text{min}\left\{E_{j}\right\}|\text{dist}\left(i,j\right)\leq k,\forall X_{ij}=1\right\}, \end{array}$$

where *E*_*j*_ represents the battery energy of sensor node *j*. To comprehensively consider the energy and sociality of sensor nodes, this study defines the weight *W*_*i*_ of each node in the region as follows:

(5) $$\begin{array}{c} W_{i}=\delta \rho_{i}+\beta \frac{\text{batt}\left(i\right)}{E_{0}}+\gamma \frac{E_{i}}{E_{0}}. \end{array}$$

Among them, *δ*, *β*, and *γ*, respectively, represent the proportion of node density, minimum battery energy, and self-energy EI of sensor node *i* in the *k*-hop range in the process of calculating the weight of each sensor node, and *δ* + *β* + *γ* = 1, 0 ≤ *δ*, *β*, *γ* ≤ 1; *E*_0_ represents the energy of all nodes in the zone where the sensor node *i* is located.

Firstly, the sensor node with the largest weight is selected as the anchor point in the region and added to the anchor point queue A; then, the *k*-hop neighbor nodes connected to the node are removed from the weighted queue, and the remaining sensor nodes are rearranged in ascending order according to the node weight; then, the node with the largest weight value in the new queue is selected as the anchor point, and the new anchor point is added to the anchor point queue.

At this time, anchor queue A is all the selected anchors in the partition, and the shortest migration path *L*_*tsp*_ of all anchors in the queue can be calculated according to the TSP problem. If *L*_*tsp*_ ≤ *L*_b_, *L*_b_ is the upper limit of the migration path of DAM, then anchor queue A is the final anchor selection queue; otherwise, the sensor node with the smallest weight in queue A is removed and the shortest migration path of queue A is recalculated until *L*_*tsp*_ meets the path constraint *L*_*b*_.

**Figure 4:**
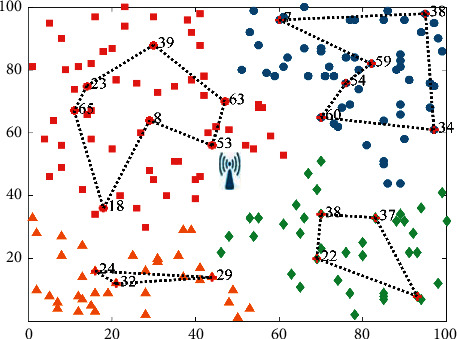
Example of anchor point selection.

Since the anchor selection scheme is related to the number and energy of neighbor nodes in its *k*-hop, *k* is set to 3 according to the setting of reference [18]. On the basis of Figure 3, 200 rechargeable sensor nodes are also selected in the uniform sensing area. Through the network zoning scheme VLD, the example results of anchor point selection in each area are shown in Figure 4. The black dotted line represents the TSP path composed of anchor points, and the points with labels represent the anchor points selected during this execution. There are four TSP paths in the figure, representing the shortest path of all anchors in each partition.

### 4.2. Data Transmission Path

Some nodes in the cluster transmit data to the anchor in the form of multi-hop static routing. Once the DAM reaches the fixed dwell time at anchor point a, it immediately goes to the next anchor point for data acquisition.

When other sensor nodes in the cluster transmit data to the anchor point, the node does not send data to all physical links connected to it, but has certain rules.

In the process of transmitting data from anchor a to DAM, anchor a is taken as the root node, and all sensor nodes within its one-hop range are regarded as the child nodes of each anchor. The child nodes of the anchor point are recorded as primary nodes, and the primary node set of each anchor point does not contain other anchor points. This process is repeated until the *k*th hop sensor node of anchor point a is calculated, that is, the *k*-th node. Each level of sensor node does not contain its parent node. At this time, the data transmission path of each sensor node is set, and each anchor point has no parent node and has only child nodes, and *k*-level nodes have only parent nodes and no child nodes. A node may belong to multiple clusters, but it does not affect the data transmission between nodes.

**Figure 5:**
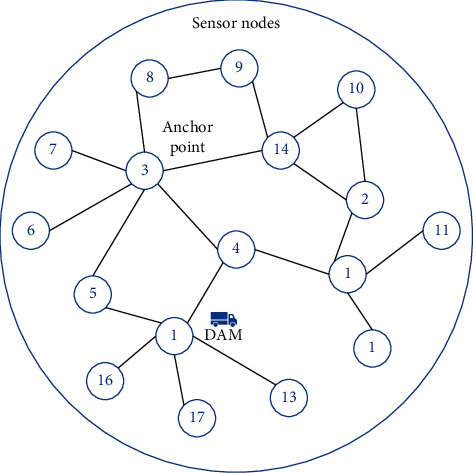
Example of network data transmission.

Figure 5 contains 17 sensor nodes, of which 3 nodes are selected as anchor points, and node numbers 1, 3, and 15 are used to obtain the data generated by the nodes in their clusters. A sensor node can transmit data to different anchors, such as node 14, which can transmit data to anchors 3 and 15 at the same time.

- When DAM stays at anchor 3, node 14 transmits data to anchor 3.
- When DAM moves to node 15, node 14 transmits data to node 2 first; then, node 2 forwards the collected data to anchor 15; anchor 15 transmits the collected data to DAM.

## 5. Optimization of Network Energy Expenditure

After determining the network subarea, anchor point, and the moving path of DAM, the next step of this study is how to minimize the network energy expenditure while collecting sensor node data when DAM moves at each anchor point. This section first introduces the energy expenditure model of a single sensor node and then defines the data acquisition performance optimization problem as the problem of minimizing network energy expenditure. According to the energy expenditure model of sensor nodes and node data transmission path, the optimal node data perception rate and link transmission rate are obtained using duality theory and gradient method. When DAM completes a round of data acquisition process, the strategy in this study will reselect the anchor points in the subarea according to the remaining battery energy of the nodes in the previous round and calculate the optimal node perception rate and link rate again through the optimization function, to achieve the optimal network performance.

### 5.1. Sensing and Data Transmission Energy Expenditure

*e *_*s*_, *e*_*r,*_ and *e*_*t,*_ respectively, represent the average energy consumed by sensor nodes to sense, receive, and send unit data. The routing between sensor nodes set in this study is static routing. Node-aware data can only be sent according to a specific route. Therefore, the energy expenditure of node-aware, received, and sent data can be expressed as a constant. Generally, sensor nodes' sensing and transmitting data account for most of the energy expenditure of nodes. Therefore, this study only considers the energy expenditure of nodes in sensing, transmitting, and receiving states and does not consider the energy expenditure in other states.

(6) $$\begin{array}{c} R_{i}^{a}=\sum_{g\in C_{i,a}}f_{gi}^{a}, \end{array}$$

where *f*_*gi*_^*a*^ represents the link rate of physical link (*g*, *i*) when DAM stays at anchor point *a*. *C*_*i*, *a*_ represents the set of child nodes of node *i* when DAM stays at anchor point *a*. Similarly, PI represents the parent node set of node *i* when DAM stays at anchor point *a*.

(7) $$\begin{array}{c} T_{i}^{a}=r_{i}^{a}+R_{i}^{a}\\ =\sum_{j\in P_{i.a}}f_{ij}^{a}. \end{array}$$

Since the above operations need to be completed in a very short time, when the DAM stays at anchor *a*, the energy expenditure of sensor node *i* is as follows:

(8) $$\begin{array}{c} P_{i}^{a}=\left(e_{s}+e_{t}\right)r_{i}^{a}+\left(e_{r}+e_{t}\right)R_{i}^{a}=e_{s}r_{i}^{a}+e_{t}\sum_{j\in P_{i.a}}f_{ij}^{a}+e_{r}\sum_{g\in C_{i,a}}f_{gi}^{a}. \end{array}$$

It can be seen from the above formula that when DAM stays at anchor point a, the energy expenditure of sensor node *i* can be divided into two parts according to the data source: the energy consumed by node sensing and transmitting its own data and the energy consumed by receiving and sensing the data of other nodes. Due to the dynamic change in physical link rate in the network, to represent the link rate of transmitting and receiving data, this study divides the energy expenditure into three parts: sensor node sensing energy expenditure, transmitting data energy expenditure, and receiving data energy expenditure.

The remaining life of a sensor node is calculated by its remaining battery energy and dynamic energy expenditure. Then, taking the distance between the sensor nodes to be charged and the remaining lifetime as the measurement index of the ordering of the nodes to be charged [3], WESM charges the nodes according to the charging queue.

### 5.2. Optimization Function

To describe the impact of the data perception rate of sensor nodes at a specific anchor point on the performance of the whole network, this study introduces an energy expenditure function *C*_*i*_^*a*^(·) as an optimization index. Compared with *r*_*i*_^*a*^, the optimization function is strictly convex, increasing, and quadratic differentiable. Based on the energy management model and data transmission model, the minimization of network energy expenditure in WMSNS can be expressed in the following form:

(9) $$\begin{array}{c} P1:\text{min}\sum_{a\in A}\sum_{i\in N_{a}^{h}}C_{i}^{a}\left(r_{i}^{a}T\right), \end{array}$$

(10) $$\begin{array}{c} \text{s}.\text{t}.\sum_{a\in A_{i}}r_{i}^{a}T⩾M_{i}, \end{array}$$

(11) $$\begin{array}{c} r_{i}^{a}+\sum_{g\in C_{i,a}}f_{gi}^{a}=\sum_{j\in P_{i.a}}f_{ij}^{a}, \end{array}$$

(12) $$\begin{array}{c} \tau_{a}\Phi \left(f,r\right)\leq E_{i}. \end{array}$$

Among them,

- *r*_*i*_^*a*^,  *f*_*ij*_^*a*^⩾0,  *r* = {*r*_*i*_^*a*^},  *f* = {*f*_*ij*_^*a*^}, indicating that the data perception rate and link transmission rate of the sensor node are not less than 0.
- Φ(*f*, *r*) = ∑_*j*∈*P*_*i*,*a*__*f*_*ij*_^*a*^*e*_*t*_ + ∑_*m*∈*C*_*i*,*a*__*f*_*mi*_^*a*^*e*_*r*_ + *r*_*i*_^*a*^*e*_*s*_, which represents the energy expenditure of sensor node *i* at a certain time.
- *τ*a=(T-Ltsp/v)/|A|; on the premise of known data acquisition cycle, cycle *T* subtracts the time consumed by DAM movement, and the remaining time is evenly distributed at each anchor point to ensure the fairness of DAM data acquisition at the anchor point.
- *N*_*a*_^*h*^ indicates the sensor node covered by anchor point *a* in the *h*-th zone, *N*_*a*_^*h*^⊆*N*^*h*^.
- M_i_ represents the minimum amount of data that sensor node *i* needs to upload to DAM in a data acquisition cycle.
- E_i_ represents the remaining battery energy of sensor node *i*.
- Formula (9) is the optimization function of network energy expenditure, and formulas (10) to (12) represent the constraints of problem *P*1.
- Formula (10) indicates that for each sensor node *i*, the total amount of data uploaded at all adjacent anchor points should not be less than the limited amount of node data uploaded. Otherwise, to minimize the energy expenditure of sensor network nodes, the amount of uploaded data of each node is directly 0.
- Formula (11) shows that the output data stream of a sensor node should be equal to the sum of its own data sensing stream and its input data stream. The formula ensures that the output flow and input flow of each sensor node are balanced, which corresponds to the set of child nodes and parent nodes of each node.
- Formula (12) ensures that within a data acquisition cycle *T*, the energy expenditure of sensor nodes for communication (sensing, transmitting, and receiving data) shall not be greater than the remaining battery energy of the node, which prolongs the life of the node to the greatest extent and provides a sufficient condition for the long-term operation of WMSNS; through the above description, it is found that problem *P*1 is a convex optimization problem. Because of its strict convexity, the solution *r*_*i*_^*a*^ of the problem is unique. If the energy expenditure equation *C*_*i*_^*a*^(·) is a linear equation, it does not have strict convexity, so a quadratic regular term is added to the optimization equation *ε*∑_*i*_∑_*a*_(*r*_*i*_^*a*^*T*)^2^, so that the optimization function is strictly convex and the solution of the problem is unique, where *ε* is an extremely small constant term, making the quadratic regular term produce relatively small changes each time [24]. To ensure the fairness of the network and the strict convexity of the energy expenditure equation, *C*_*i*_^*a*^(*x*_*i*_^*a*^*T*) = *ω*_*i*_^*a*^(*x*_*i*_^*a*^*T*)^2^ is defined in this study.

Using the Lagrange multiplier method to solve optimization problems, the corresponding Lagrange function of *P*1 can be defined as follows:

(13) $$\begin{array}{c} L\left(r,f,\mu ,\lambda ,\alpha \right)=\sum_{a\in A}\sum_{i\in N_{a}^{h}}C_{i}^{a}\left(r_{i}^{a}T\right)-\sum_{i\in N}\mu_{i}\left(\sum_{a\in A_{i}}r_{i}^{a}T-M_{i}\right)\\ +\sum_{a\in A}\sum_{i\in N_{a}^{h}}\lambda_{i}^{a}\left(r_{i}^{a}+\sum_{g\in C_{i,a}}f_{gi}^{a}-\sum_{j\in P_{i.a}}f_{ij}^{a}\right)\\ +\sum_{a\in A}\sum_{i\in N_{a}^{h}}\alpha_{i}^{a}\left(\sum_{j\in P_{i,a}}f_{ij}^{a}e_{t}+\sum_{m\in C_{i,a}}f_{mi}^{a}e_{r}+r_{i}^{a}e_{s}-\frac{E_{i}}{\tau_{a}}\right). \end{array}$$

Lagrange's dual function is the minimum value of formula (9) in *r*_*i*_^*a*^, and then, the objective function of the dual function is as follows:

(14) $$\begin{array}{c} D\left(\mu ,\lambda ,\alpha \right)=\underset{r}{\text{min}}L\left(r,f,\mu ,\lambda ,\alpha \right). \end{array}$$

Lagrange dual function can be defined as follows:

(15) $$\begin{array}{c} \text{max}D\left(\mu ,\lambda ,\alpha \right)=\text{max}\underset{r}{\text{min}}L\left(r,f,\mu ,\lambda ,\alpha \right). \end{array}$$

Let *r*^*∗*^={*r*_*i*_^*a*^ ≥ *|a* ∈ *A*, *i* ∈ *N*_*a*_^*h*^} and *f*^*∗*^={*f*_*ij*_^*a*^*|a* ∈ *A*, *i* ∈ *N*_*a*_^*h*^, *j* ∈ *P*_*i*,*j*_^*a*^} be the optimal solution of problem *P*1. The Lagrange function can be decomposed into two subproblems: node rate control problem and routing scheduling problem. *L* (*r*, *f*, *μ*, *λ*, *α*) is removed. The constant term can be divided into two sub-function forms:

(16) $$\begin{array}{c} L_{1}\left(r,\mu ,\lambda ,\alpha \right)=\sum_{a\in A}\sum_{i\in N_{a}^{h}}C_{i}^{a}\left(r_{i}^{a}T\right)-\sum_{i\in N}\sum_{a\in A_{i}}r_{i}^{a}T\mu_{i}+\sum_{a\in A}\sum_{i\in N_{a}^{h}}\lambda_{i}^{a}r_{i}^{a}+\sum_{a\in A}\sum_{i\in N_{a}^{h}}\alpha_{i}^{a}r_{i}^{a}e_{s}\\ =\sum_{a\in A}\sum_{i\in N_{a}^{h}}\left(C_{i}^{a}\left(r_{i}^{a}T\right)-r_{i}^{a}T\mu_{i}+\lambda_{i}^{a}r_{i}^{a}+\alpha_{i}^{a}r_{i}^{a}e_{s}\right), \end{array}$$

(17) $$\begin{array}{c} L_{2}\left(f,\lambda ,\alpha \right)=\sum_{a\in A}\sum_{i\in N_{a}^{h}}\lambda_{i}^{a}\left(\sum_{g\in C_{i,a}}f_{gi}^{a}-\sum_{j\in P_{i.a}}f_{ij}^{a}\right)+\sum_{a\in A}\sum_{i\in N_{a}^{h}}\alpha_{i}^{a}\left(\sum_{j\in P_{i,a}}f_{ij}^{a}e_{t}+\sum_{m\in C_{i,a}}f_{mi}^{a}e_{r}\right)\\ =\sum_{a\in A}\sum_{i\in N_{a}^{h}}\left(\lambda_{i}^{a}\sum_{g\in C_{i,a}}f_{gi}^{a}+\alpha_{i}^{a}\sum_{m\in C_{i,a}}f_{mi}^{a}e_{r}\right)+\sum_{a\in A}\left(\sum_{i\in N_{a}^{h}}\alpha_{i}^{a}\sum_{j\in P_{i,a}}f_{ij}^{a}e_{t}-\sum_{i\in N_{a}^{h}}\lambda_{i}^{a}\sum_{j\in P_{i.a}}f_{ij}^{a}\right)\\ =\sum_{a\in A}\sum_{i\in N_{a}^{h}}\left(\underset{g\in C_{i,a}}{\sum }f_{gi}^{a}\left(\lambda_{i}^{a}+\alpha_{i}^{a}e_{r}\right)+\underset{j\in P_{i,a}}{\sum }f_{ij}^{a}\left(\alpha_{i}^{a}e_{t}-\lambda_{i}^{a}\right)\right). \end{array}$$

For the problem of data perception rate of sensor node *i*, since *C*_*i*_^*a*^ is a monotonically increasing function, to obtain the minimum energy expenditure of node *i*, the objective function can be realized only when formula (10) is equal. Therefore, the problem of minimizing the energy expenditure of a single sensor node *i* can be defined as follows:

(18) $$\begin{array}{c} P2:\begin{array}{cc} \text{min} & \sum_{a\in A_{i}}C_{i}^{a}\left(r_{i}^{a}T\right)+\sum_{a\in A_{i}}q_{i}^{a},\\ \text{s.t.} & \sum_{a\in A_{i}}r_{i}^{a}T⩾M_{i}, \end{array} \end{array}$$

where *r*_*i*_^*a*^ ≥ 0,  ∀ *a* ∈ *A*_*i*_. Let *x*_*i*_^*a*^=*r*_*i*_^*a*^*T*,  *q*_*i*_^*a*^=*σ*_*i*_^*a*^*x*_*i*_^*a*^,  *q*_*i*_^*a*^ be the energy that sensor node *i* needs to pay to obtain data transmission opportunities when DAM stays at anchor point a, *σ*_*i*_^*a*^ be the energy expenditure of transmitting single data, and *x*_*i*_^*a*^ be the data transmission amount of sensor node *i* at anchor a. The energy expenditure of sensor node *i* can be divided into two parts: (1) the consumption of transmitting data to its neighbor anchor and (2) energy requirements for obtaining data transmission opportunities. The energy expenditure of sensor node *i* can be adjusted dynamically by adjusting its payment energy independently.

Introducing a new Lagrange multiplier *υ*_*i*_ constructs the Lagrange function of problem *P*2 and solves it through the Karush–Kuhn–Tucker (KKT) condition:

(19) $$\begin{array}{c} \left.\begin{array}{c} \frac{1}{\sigma_{i}^{a}}C_{i}^{\prime a}\left(\frac{q_{i}^{a}}{\sigma_{i}^{a}}\right)+1-\frac{\nu_{i}}{\sigma_{i}^{a}}=0\\ \nu_{i}\left(\sum_{a\in A}\frac{q_{i}^{a}}{\sigma_{i}^{a}}-M_{i}\right)=0 \end{array}\right\}. \end{array}$$

Through KKT conversion of formula (13) and comparison with (19), the Lagrange multiplier can be obtained; *μ*_*i*_ = *υ*_*i*_. Let *f*_i_ represent the objective function of minimizing the energy expenditure of sensor node *i*, that is, *P*2. For each node *i*, if ${\hat{a}}_{i}$ represents the anchor point with the lowest cost of node *i*, then

(20) $$\begin{array}{c} {\hat{a}}_{i}=\text{argmin}\left\{\frac{\partial f_{i}\left(r_{i}\right)}{\partial r_{i}^{a}}\right\}\\ =\text{argmin}\left\{C_{i}^{\prime a}\left(r_{i}^{a}T\right)T-T\mu_{i}+\lambda_{i}^{a}+\alpha_{i}^{a}e_{s}\right\}. \end{array}$$

If there are multiple minimum cost anchors for sensor node *i*, one anchor can be randomly selected as the minimum cost anchor. Through the adaptive algorithm [24], we can get the following:

(21) $$\begin{array}{c} q_{i}^{a}=\left\{\begin{array}{cc} 0, & \mu_{i}<C_{i}^{\prime a}\left(0\right)+\sigma_{i}^{a},\\ \sigma_{i}^{a}{\left(C_{i}^{\prime a}\right)}^{-1}\left(\mu_{i}-\sigma_{i}^{a}\right), & \mu_{i}\geq C_{i}^{\prime a}\left(0\right)+\sigma_{i}^{a}. \end{array}\right. \end{array}$$

By adjusting the size of *q*_*i*_^*a*^, the data perception rate of nodes can be changed, to minimize the network energy expenditure. For nodes *i*, $a\in A_{i},a={\hat{a}}_{i}$, the data transmission volume at anchor point a can be increased by increasing the value of *q*_*i*_^*a*^. Accordingly, the data transmitted at other anchor points will be reduced, and finally, the energy expenditure of sensor node *i* will be reduced; otherwise, for node *i*, $a\in A_{i},a\neq {\hat{a}}_{i}$, reducing the value of *q*_*i*_^*a*^ will increase the amount of data transmission at the least cost anchor. Here, assume that the *σ*_*i*_^*a*^ of sensor node *i* is a fixed value.

Through dual transformation, formula (17) can be transformed into

(22) $$\begin{array}{c} \underset{f}{\max}\sum_{a\in A}\sum_{i\in N_{a}^{h}}\left(\sum_{g\in C_{i,a}}f_{gi}^{a}\left(\lambda_{i}^{a}+\alpha_{i}^{a}e_{r}\right)+\sum_{j\in P_{i,a}}f_{ij}^{a}\left(\alpha_{i}^{a}e_{t}-\lambda_{i}^{a}\right)\right)\\ =\sum_{a\in A}\sum_{i\in N_{a}^{h}}\sum_{j\in P_{i,a}}\left(\lambda_{j}^{a}+\alpha_{j}^{a}e_{r}+\alpha_{i}^{a}e_{t}-\lambda_{i}^{a}\right)f_{ij}^{a}. \end{array}$$

The constraints are formulas (12) and (13). To solve the routing scheduling problem, we can start from the sensor node with empty subset and calculate the optimal value of the Lagrange multiplier of the node according to its own Lagrange multiplier initial value and sub-gradient method. Then, the node transmits the optimal value to the neighbor node according to the physical link. The neighbor node obtains its own optimal Lagrange multiplier value by obtaining the optimal multiplier value. This process is repeated until the optimal multiplier value of all nodes is obtained. Through this method, the optimal physical link transmission rate of sensor node *i* can be obtained, which is actually equivalent to finding the set:

(23) $$\begin{array}{c} X_{i}=\left\{\left(j,a\right)|\lambda_{j}^{a}+\alpha_{j}^{a}e_{r}+\alpha_{i}^{a}e_{t}-\lambda_{i}^{a}>0,\forall j\in P_{ij}^{a},\forall a\in A\right\}. \end{array}$$

The parent node and anchor point corresponding to the maximum value in the sensor node *i* set *X*_*i*_ are selected one by one, and the maximum link rate *f*_*ij*_^*a*^=(*E*_*i*_/*τ*_*a*_)*e*_*t*_ is assigned, and the remaining battery energy of the node is updated at the same time. This process is repeated until the collection is empty. In addition, the heuristic distribution algorithm [31] can be used to solve such problems.

The neighbor node then calculates its own data perception rate and link transmission rate. The update rule is as follows:

(24) $$\begin{array}{c} \left.\begin{array}{c} \mu_{i}\left(t+1\right)={\left[\mu_{i}\left(t\right)+\eta \left(M_{i}-\sum_{a\in A_{i}}r_{i}^{a}T\right)\right]}^{+}\\ \lambda_{i}^{a}\left(t+1\right)={\left[\lambda_{i}^{a}\left(t\right)+\eta \left(r_{i}^{a}+\sum_{g\in C_{i,a}}f_{gi}^{a}-\sum_{j\in P_{i,a}}f_{ij}^{a}\right)\right]}^{+}\\ \alpha_{i}^{a}\left(t+1\right)={\left[\alpha_{i}^{a}\left(t\right)+\eta \left(r_{i}^{a}e_{s}+\sum_{g\in C_{i,a}}f_{gi}^{a}e_{r}+\sum_{j\in P_{i,a}}f_{ij}^{a}e_{t}-\frac{E_{i}}{\tau_{a}}\right)\right]}^{+} \end{array}\right\}, \end{array}$$

where *t* is the number of iterations, *η* is the iteration step size, and [·]^+^ = max{·, 0}. In this study, the Lagrange multipliers of all sensor nodes are used, *μ*_i_, *λ*, and *α*. The initial values of are set to 1. Since *C*_*i*_^*a*^(·) is a strictly convex function, *f*_*ij*_^*a*^ has a unique maximum when the multiplier is a unique definite value.

Because the routing scheduling problem is linear, the optimal value obtained by Lagrange cannot be directly applied to the original problem. Therefore, this study uses the method in reference [32] to restore the original solution of *f*_*ij*_^*a*^. Once the original solution $\left\{{\hat{f}}_{ij}^{a}\right\}$ converges, *f*_*ij*_^*a*^ and *r*_*i*_^*a*^ are the optimal solutions of the optimization function P1.

## 6. Experimental Verification and Analysis

This section will verify the efficiency of the proposed scheme and the overall network strategy through a large number of experiments. All experimental results are calculated by MATLAB. Firstly, the convergence of sensor node data perception rate based on minimizing network energy expenditure function is verified. Secondly, by comparing the amount of data collected by the base station under different zoning schemes, the efficiency of the zoning scheme VLD is verified; then, because the cruise path length when DAM collects anchor data is closely related to the energy expenditure of the network system, the efficiency of the anchor selection scheme ASS in this study is verified by calculating the moving length of DAM in three different anchor selection schemes. To further verify the impact of anchor selection scheme on the performance of the whole network, this study analyzes the network life cycle under different anchor selection schemes based on the number of surviving nodes in the network and the amount of data collected by the base station. Finally, the performance of the overall strategy is verified: the efficiency of this strategy by comparing the data volume obtained by the base station, energy expenditure, and the impact of charging efficiency on energy expenditure under different schemes is comprehensively verified; by comparing the overall performance under different topology schemes, the stability of this strategy is verified.

The experimental scenario is shown in Figure 3. 200 rechargeable sensor nodes are evenly distributed at 100 m × within 100 m sensing area. The sensing range of each sensor node is the same, and all nodes use the same hardware, i.e., energy receiving device and power storage device (see Table 1 for network parameters). According to [16], there are three adjustable parameters in the anchor point selection scheme *δ*, *β*, and *γ*, and set to 0.4, 0.3, and 0.3, respectively.

**Figure 6:**
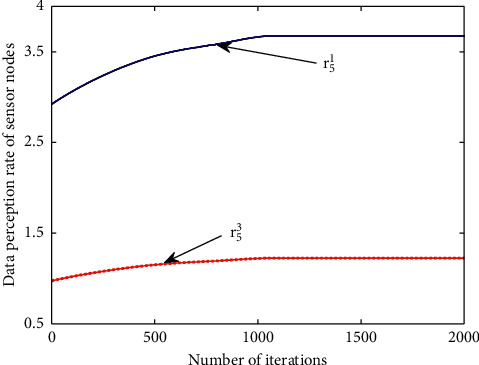
Data perception rate of sensor nodes.

The data perception rate *r*_*i*_^*a*^ obtained by minimizing the network energy optimization function is shown in Figure 6. It shows the change in the data perception rate *r*_5_^1^ at anchor 1 and *r*_5_^3^ at anchor 3 of sensor node 5 with the increase in the number of iterations. In the initial operation stage, the battery power of the sensor node is determined using the random number generated. It can be seen from the figure that the data perception rates *r*_5_^1^ and *r*_5_^3^ fluctuate greatly at the beginning; after about 1000 iterations, the data perception rates *r*_5_^1^ and *r*_5_^3^ tend to be stable and finally reach the convergence state and the optimal value. This is because in the initial stage, with the change in Lagrange multiplier and the change in physical link transmission rate, the dynamic change in these values has a great impact on the data perception rate *r*_*i*_^*a*^ of sensor nodes. With the increase in the number of cycles and the gradual decrease in the iteration step of Lagrange multiplier, the changes in multiplier and link rate are also gradually shrinking, and the impact on the optimal value of data perception rate is becoming smaller and smaller, so that the data perception rate of nodes finally reaches a stable state.

**Figure 7:**
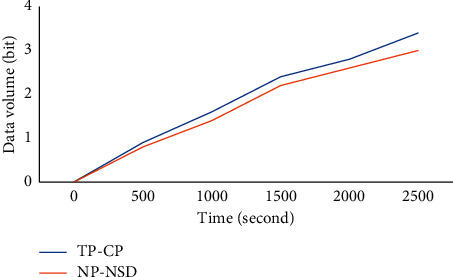
Comparison of data collected under TP-CP and VLD schemes.

Figure 7 shows the data acquisition volume of two network partition schemes VLD and TP-CP [16] over time. TP-CP adopts the secondary zoning method based on the central point, which is mainly divided according to the distance from the sensor node to the central node and the static routing length. As can be seen from the figure, with the increase in time, the amount of data collected by the base station in the two schemes is more and more. However, VLD is slightly better than TP-CP. This is because VLD divides the network according to the vicinity likeness of sensor nodes and the distance between nodes. In this way, there are fewer physical link connections between nodes in the divided areas, and considering the distance factor, the nodes closer to each other are likely to be divided into the same area. TP-CP only considers the routing length and distance from the sensor node to the central node. Therefore, TP-CP may disconnect many links between regions, which increases the communication energy expenditure of node data transmission to the anchor point. Although the route from the node to the central node is small, it cannot guarantee the length of the route to the anchor point, which is well guaranteed by the vicinity likeness of VLD. Therefore, in the same scenario, VLD has better performance than TP-CP.

**Figure 8:**
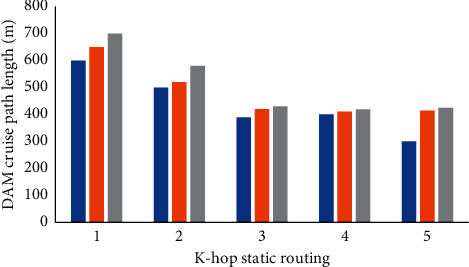
Influence of anchor coverage *k* on DAM cruise length.

Then, the same zoning scheme VLD is used to compare the cruising path length of DAM with three anchor point selection schemes ASS, AS-NAE [16], and AS-LE [17]. The results are shown in Figure 8.

AS-NAE selects the anchor point according to the number of *k*-hop neighbor nodes and the minimum power of nodes in the area. The AS-LE selects the anchor point only according to the power of the *k*-hop neighbor node. It can be seen from the figure that with the increase in anchor point coverage *k*, the cruise path of DAM gradually decreases. This is because with the increase in cluster length, the coverage of each anchor point also increases, which reduces the number of anchor points and the cruise path of DAM. In addition, the length of ASS is the shortest, followed by AS-NAE, and AS-LE is the longest. This is because AS-LE focuses on the minimum battery power within *k*-hops of each node. There may be some nodes whose minimum battery energy is the same node. At this time, a node is randomly selected as the anchor point. The selected anchor point may be located at the boundary of the region, which increases the cruising length of DAM. Compared with the other two schemes, ASS not only considers the number of neighbors within the k-hop of the sensor node and the minimum battery power but also takes into account the energy of the node itself, comprehensively selects the node with more power and better sociality as the anchor point, avoids the influence of the energy of the node itself in AS-NAE, and selects the remote node as the anchor point. Therefore, the ASS scheme in this study is better.

**Figure 9:**
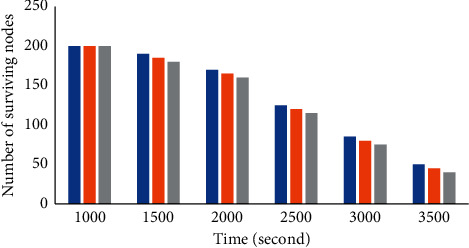
Comparison of the number of surviving sensor nodes under the three schemes of ASS, AS-NAE, and AS-LE.

Figure 9 shows the number of surviving nodes of three adaptive anchor selection schemes ASS, AS-NAE, and AS-LE at different running times. It can be seen from the figure that the overall trend of the number of surviving sensor nodes is less and less with the operation of time. However, in different time periods, the number of surviving nodes under ASS scheme is greater than that of AS-NAE and AS-LE. With the operation of time, AS-NAE and AS-LE do not consider that the energy expenditure rate will increase due to more data sent and received by the anchor point; accordingly, the probability of anchor death is higher. While ASS considers the energy of neighborhood nodes and the energy of the anchor itself, it avoids the relatively low energy of the selected anchor to the greatest extent. Compared with AS-NAE and AS-LE, ASS can effectively increase the service time of anchor points and reduce the frequency of energy hot spots. Therefore, the node survival rate in ASS scheme is higher.

**Figure 10:**
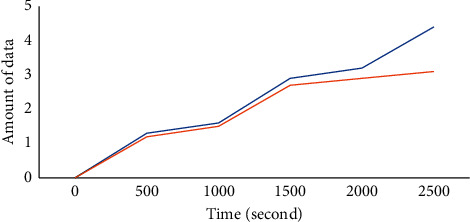
Comparison of data collected under ASS and AS-NAE schemes.

Figure 10 shows the amount of data transmitted from the sensor node to the base station under the two anchor selection schemes ASS and AS-NAE. From the perspective of fairness, this study selects [16] with the same application scenario as a comparison and tests the amount of data collected under the two anchor selection schemes ASS and AS-NAE. On the premise of using the same network partition scheme and constructing the same optimization function, given a fixed network energy expenditure threshold, the amount of data collected by the two schemes increases with the increase in time, but the anchor selection scheme ASS in this study can obviously enable the base station to obtain more data. This is because ASS fully considers the influence of the sensor anchor. In the process of network operation, sensor nodes with higher battery energy and more neighbor nodes are more likely to be selected as anchor points. In AS-NAE, the sensor node as the anchor point will die due to energy depletion to a large extent. At this time, if the more remote node is selected as the anchor point, the sociality of the node will not necessarily be weakened, and the communication energy expenditure of other nodes will increase, resulting in relatively less data collected by the base station. Therefore, the performance of ASS scheme is better.

**Figure 11:**
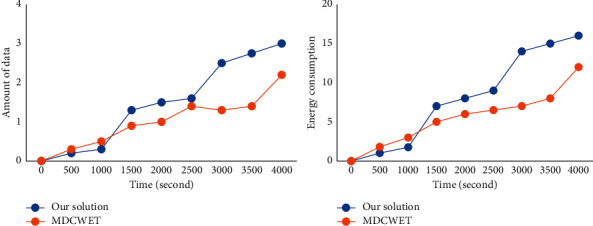
Performance comparison between the overall strategy and MDCWET in this study.

Figure 11 shows the performance comparison between the overall strategy of this study and MDCWET [16] from two aspects: the amount of data collected by the base station and energy expenditure. It can be seen from the figure that with the operation of time, the amount of data collected by the base station under the two overall strategies is increasing. In the early networking stage, the amount of data collected by this strategy is small, but after the networking is stable, the amount of data obtained by this strategy is significantly greater than that of MDCWET. With the increase in time, the data gap between this strategy and MDCWET is becoming larger and larger. This is because the number of dead sensor nodes in MDCWET increases with the operation of time, resulting in a sharp reduction in the amount of data generated in the network. However, when using the strategy in this study, the difference in the amount of data collected by the base station does not decrease significantly. In the comparison of energy expenditure between the overall strategy and MDCWET in this study, the energy expenditure produced by the two strategies shows an increasing trend with the operation of time. After stable networking, the energy expenditure of this strategy is significantly higher than that of MDCWET. This is because the energy expenditure in the network is mainly caused by the anchor point, and the energy expenditure of the anchor point has a positive relationship with the amount of data transmitted. Therefore, the larger the amount of data, the greater the energy expenditure generated by the network. It can be seen that this strategy can effectively help the system increase the amount of data acquisition and improve the efficiency of data acquisition.

**Figure 12:**
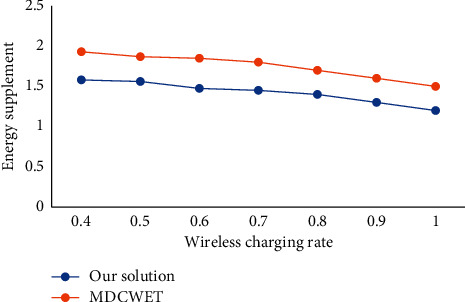
Comparison of influence of wireless energy supplement efficiency on energy expenditure.

Figure 12 shows the effect of wireless energy supplement efficiency on energy expenditure It can be seen that the higher the charging efficiency, the smaller the energy expenditure in the network, and it remains stable when the charging efficiency is high. The energy expenditure rate of the overall strategy proposed in this study is significantly lower than that of MDCWET. This is because this study considers the residual energy of the anchor when determining the anchor and timely serves the anchor that needs to be charged, which reduces the energy expenditure of the anchor and the energy expenditure of the whole network. It can be seen that the overall strategy of this study can effectively reduce the energy expenditure in the network and charge the nodes efficiently.

**Figure 13:**
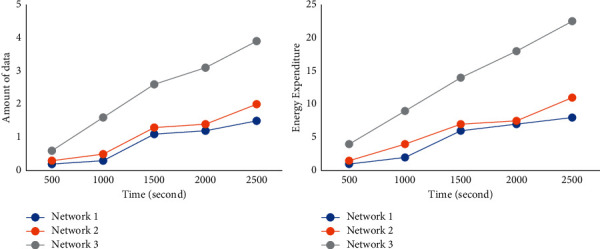
Performance comparison of the overall strategy in this study under three different topologies.

Figure 13 shows the amount of data and energy expenditure collected by the base station under different topologies. It can be seen from the figure that with the operation of time, the amount of data collected by the base station continues to increase, and the overall growth is relatively stable. This is because the anchor selection strategy adopted in this study can obtain the data stored by the node in time, reasonably arrange the moving path of DAM, and complete the data acquisition. The energy expenditure of nodes increases with the operation of time and is close to the growth trend of data volume. This is because the main energy expenditure in the network comes from the anchor point. The increase in data volume leads to an increase in anchor energy expenditure, which leads to an increase in overall energy expenditure. It can be seen that the strategy in this study can be stably applied to different topologies.

## 7. Conclusion

The contributions of this study are as follows: a network partition scheme based on vicinity likeness and distance of sensor nodes is designed to divide the whole sensor network into multiple subregions; this study proposes ASS solution to obtain the anchor in the subregion. The optimization problem of wireless multimedia sensor network is decomposed into several sub-dual problems using the dual problem decomposition theory to obtain the global optimal solution; through a large number of experimental results, the efficiency of the proposed VLD, ASS, and the overall network strategy is verified. The experimental results show that the proposed scheme and strategy not only ensure the continuous operation of the network but also achieve better network performance.

This study studies the efficient data acquisition and the optimization of the overall energy expenditure of the network in WMSNS. In the next step, the impact of the multifunctional mobile car on the network performance will be considered; that is, the car has the functions of energy supply and data acquisition at the same time. The mobile path of the multifunctional car and the performance comparison with the single-function car in different scenarios will be studied.

## Figures and Tables

**Table 1:** Parameter setting.

Name	Value
E_0_	1 J
e_s_	0.05 mJ
e_r_,e_t_	0.3 mJ
*v*	3 m/s∼5 m/s

## Data Availability

The data used to support the findings of this study are available from the corresponding author upon request.
